# Peri-Transplant Inflammation and Long-Term Diabetes Outcomes Were Not Impacted by Either Etanercept or Alpha-1-Antitrypsin Treatment in Islet Autotransplant Recipients

**DOI:** 10.3389/ti.2024.12320

**Published:** 2024-01-31

**Authors:** Tasneem R. Abdel-Karim, James S. Hodges, Kevan C. Herold, Timothy L. Pruett, Karthik V. Ramanathan, Bernhard J. Hering, Ty B. Dunn, Varvara A. Kirchner, Gregory J. Beilman, Melena D. Bellin

**Affiliations:** ^1^ Department of Pediatrics, University of Minnesota, Minneapolis, MN, United States; ^2^ Division of Biostatistics, School of Public Health, University of Minnesota, Minneapolis, MN, United States; ^3^ Departments of Immunobiology and Internal Medicine, Yale University, New Haven, CT, United States; ^4^ Department of Surgery, University of Minnesota, Minneapolis, MN, United States; ^5^ Department of Surgery, Medical College of Wisconsin, Milwaukee, WI, United States; ^6^ Department of Surgery, Stanford University, Palo Alto, CA, United States

**Keywords:** islet transplantation, diabetes, beta cell death, anti-inflammatory, TPIAT

## Abstract

The instant blood-mediated inflammatory response (IBMIR) causes islet loss and compromises diabetes outcomes after total pancreatectomy with islet autotransplant (TPIAT). We previously reported a possible benefit of etanercept in maintaining insulin secretion 3 months post-TPIAT. Here, we report 2-year diabetes outcomes and peri-operative inflammatory profiles from a randomized trial of etanercept and alpha-1 antitrypsin (A1AT) in TPIAT. We randomized 43 TPIAT recipients to A1AT (90 mg/kg IV x6 doses, *n* = 13), etanercept (50 mg then 25 mg SQ x 5 doses, *n* = 14), or standard care (*n* = 16). Inflammatory cytokines, serum A1AT and unmethylated insulin DNA were drawn multiple times in the perioperative period. Islet function was assessed 2 years after TPIAT with mixed meal tolerance test, intravenous glucose tolerance test and glucose-potentiated arginine induced insulin secretion. Cytokines, especially IL-6, IL-8, IL-10, and MCP-1, were elevated during and after TPIAT. However, only TNFα differed significantly between groups, with highest levels in the etanercept group (*p* = 0.027). A1AT increased after IAT in all groups (*p* < 0.001), suggesting endogenous upregulation. Unmethylated insulin DNA ratios (a marker of islet loss) and 2 years islet function testing were similar in the three groups. To conclude, we found no sustained benefit from administering etanercept or A1AT in the perioperative period.

## Graphical Abstract

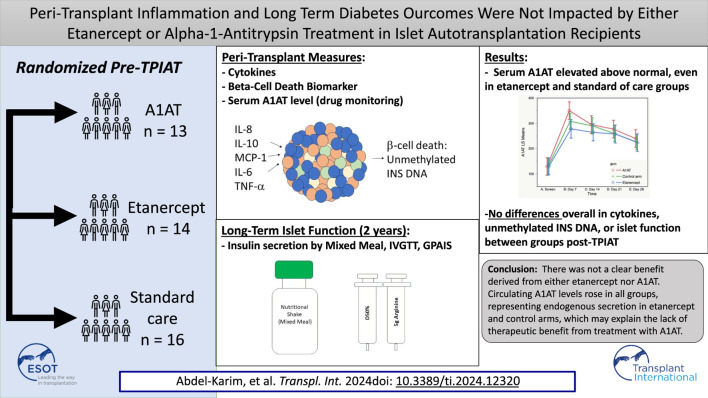

## Introduction

Total pancreatectomy (TP) is an effective treatment to relieve or reduce severe pain in patients with chronic pancreatitis or recurrent acute pancreatitis who do not respond to medical or endoscopic therapies. Islet auto transplantation (IAT) prevents or ameliorates brittle diabetes after total pancreatectomy [1–6].

Long-term insulin independence is an important goal in TPIAT as it reduces the burden of diabetes management and improves quality of life [4]. Many patients, however, never reach insulin independence. In one report, only 30% of patients undergoing TPIAT achieved insulin independence 3 years post-TPIAT [5]; this percentage showed a downward trend over time, dropping to about 11% 10 years post-TPIAT [7]. Successful engraftment of transplanted islet cells into the liver is crucial for long-term insulin independence. The survival and function of transplanted islet cells is negatively impacted by inflammation in the peri-transplant period, the so-called instant blood mediated inflammatory reaction (IBMIR) [8]. Therefore, blockading the innate inflammatory response could substantially improve engrafted islet mass and thereby reduce diabetes risk after TPIAT [9].

Aiming to reduce islet loss from IBMIR, we studied two promising anti-inflammatory therapies: the TNFα inhibitor etanercept, and alpha-1 antitrypsin (A1AT). Etanercept is a potent anti-inflammatory drug that targets TNFα, a key central inflammatory mediator in beta loss after transplant. Etanercept is widely used as an anti-inflammatory drug in intraportal *allo*transplantation for type 1 diabetes, with studies suggesting efficacy in this setting [10]. A1AT is a serine protease inhibitor indicated for treating alpha-1 antitrypsin deficiency. Multiple preclinical studies, including autologous islet transplant models in non-human primates, suggested that A1AT enhances islet engraftment and prevents beta cell apoptosis by suppressing the instant blood mediated inflammatory reaction [11–15]. We previously reported early outcomes with both drug therapies in a randomized pilot clinical trial, which suggested a greater first phase insulin response at 3 months only in the etanercept group [16]. It was not clear, however, whether any benefit was sustained 1 year post-TPIAT.

This current analysis reports 2-year outcomes for insulin use and islet function following TPIAT in our clinical trial participants treated with etanercept and A1AT. We also evaluated potential mechanistic pathways targeted by these agents by measuring cytokine levels, beta cell death measured by unmethylated insulin DNA, and circulating levels of A1AT during the peri-transplant period.

## Patients and Methods

### Participants

Forty-four adult patients between 18 and 68 years old, who were scheduled for TPIAT at the University of Minnesota (UMN) from 12/2016 through 3/2020, were enrolled. Exclusions included pre-existing diabetes or other medical contraindications that could compromise participant safety; please refer to our first publication from this study for details about the exclusion criteria [16]. One individual did not meet inclusion criteria for randomization based on labs obtained at the screening visit, so 43 participants were randomized to receive etanercept (*n* = 14), A1AT (*n* = 13), or no treatment (controls, *n* = 16) as detailed below.

Informed consent was obtained from all participants before screening. The study protocol was reviewed and approved by the University of Minnesota’s Institutional Review Board. This study was performed under an Investigational New Drug Application (IND #119828) from the Food and Drug Administration and registered on clinicaltrials.gov (NCT#02713997).

### Surgical and Islet Isolation Procedure

Participants underwent total pancreatectomy with partial duodenectomy, splenectomy, cholecystectomy, and roux-en-Y duodenojejunostomy [17]. Islet cells were extracted through enzymatic digestion using Vitacyte CIzyme Collagenase HA (Vitacyte LLC, Indianapolis, IN) with SERVA/Nordmark Neutral Protease NB (SERVA Electrophoresis GmbH, Heidelberg, Germany) followed by mechanical disruption using the semi-automated Ricordi method [18]. These isolated islet cells were then introduced into the liver’s portal vein. Throughout the infusion of islet cells, portal pressures were closely monitored. In cases where elevated portal pressures were observed, some islets were directed to other locations, primarily within the peritoneal cavity. Heparin was administered at the time of islet infusion in the form of a 70 unit/kg bolus, with 35 u/kg incorporated into the islet preparation and 35 u/kg given to the patient. Subsequently, low dose heparin was administered either intravenously or subcutaneously (enoxaparin) and continued for 1 week post-transplantation. Islet mass was quantified as islet equivalents (IEQ) or IEQ per kilogram of recipient body weight (IEQ/kg), which standardizes islet mass to a size of 150 μm, consistent with established practices in islet research. We also evaluated islet number (IN) and IN/kg as measures of the total count of islets without adjusting for islet volume.

### Treatment Provided

Patients were randomized in a 1:1:1 ratio to A1AT, or etanercept, or standard care. Alpha-1 antitrypsin (Aralast NP) was administered intravenously at a dose of 90 mg/kg, with the first dose administered 1 day before surgery and subsequent doses on days 3, 7, 14, 21, and 28 after infusion. Etanercept was given at a dose of 50 mg subcutaneously on day 0 (pre-operatively), and 25 mg subcutaneously on days 3, 7, 10, 14, 21.

Randomization was stratified on BMI (< or ≥27 kg/m^2^). The investigational pharmacist dispensed study medication according to a randomization schedule provided by the biostatistician. Because the drugs are administered intravenously and subcutaneously, this pilot study was not blinded.

### Study Visits and Assessments

Participants attended 2-day study visits to assess islet function before surgery (“Baseline”), at 3 months and at 1 and 2 years after TPIAT. Multiple blood draws were performed in the perioperative period for mechanistic assays, as detailed below. All study participants contributed to the mechanistic data, while 42 had remote or in-person follow up (32 in person, with metabolic testing). The “2 years” visit was complicated by COVID restrictions and an institutional-mandated pause in study visits so the 2-year window was expanded to 2–3 years post-TPIAT to capture participants who were able and willing to return. The average time of this visit was 2.2 years post TPIAT. For participants unwilling or unable to travel back, as much data as possible were collected virtually.

At each study visit, participants underwent comprehensive metabolic testing with mixed meal tolerance testing (MMTT), intravenous glucose tolerance testing (IVGTT), and glucose-potentiated arginine-induced insulin secretion (GPAIS) studies as described below. Hemoglobin A1c (HbA1c) level was also measured. Efficacy of islet graft function was assessed based on metabolic testing measures, HbA1c, insulin use and insulin dose. Blinded continuous glucose monitoring (iPro2, Medtronic) data were collected for 6 days following the 3-month, 1-year and 2-year visits to assess mean glucose, standard deviation, and percent of time in hypo- and hyperglycemia. Because iPro was discontinued by the manufacturer shortly before the conclusion of the study, CGM is available for fewer participants.

#### Mixed Meal Tolerance Testing

For the mixed-meal tolerance test (MMTT), measurements of glucose and C-peptide levels were taken at time 0, 30, 60, 90, and 120 min. Participants were given Boost HP, at a dose of 6 mL/kg (maximum 360 mL), consumed within 5 minutes after the initial time 0 blood draw. The area under the curve (AUC) for glucose (AUC glucose) and C-peptide (AUC C-peptide) were calculated using the trapezoidal rule, which included the baseline.

#### Intravenous Glucose Tolerance Testing

For the intravenous glucose tolerance test (IVGTT), a bolus of 0.3 g/kg of dextrose was administered at time 0. Samples of insulin, C-peptide, and glucose were drawn at times −10, −5, −1, and 1, 2, 3, 4, 5, 7, and 10 min. The AUC for the 10-min insulin or C-peptide values, minus the baseline, was used to determine the acute insulin response to glucose (AIRglu) and acute C-peptide response to glucose (ACRglu), respectively.

#### Glucose-Potentiated Arginine-Induced Insulin Secretion

The glucose-potentiated arginine stimulation (GPAIS) test was conducted after the IVGTT. A 20% dextrose solution was administered via infusion, commencing at +20 min after the IVGTT dextrose bolus. The infusion was maintained at a variable rate to attain a blood glucose target of approximately 230 mg/dL until the test was completed. Blood glucose levels were monitored every 5 minutes, using a bedside autoanalyzer to maintain glucose within the targeted range. At +60 min, after a minimum of 30 min of maintaining the target blood glucose level, baseline samples for glucose, insulin, and C-peptide were drawn at three intervals over 5 min. A 5-gram bolus of arginine was then administered, and samples for glucose, insulin, and C-peptide were taken at 2, 3, 4, 5, 7, and 10 min following the arginine bolus. The test results were used to calculate the glucose-potentiated acute insulin response to arginine (AIRpot) and glucose-potentiated acute C-peptide response to arginine (ACRpot) as surrogate markers for islet mass.

### Sample Collection

Inflammatory cytokines were measured at various intervals throughout the study: twice during pre-operative screening, once immediately before islet infusion, and then at 1, 3, 6, 12, 24 h, and 3 and 7 days after IAT. These cytokines included interferon gamma-induced protein 10 (IP-10), interleukin 1 alpha (IL-1 α), interleukin 1 beta (IL-1 β), interleukin 6 (IL-6), interleukin 8 (IL-8), interleukin 10 (IL-10), monocyte chemoattractant protein-1 (MCP-1) and tumor necrosis factor alpha (TNF-α).

Levels of serum Alpha 1 antitrypsin (sA1AT) were assessed at five times: before surgery and at 7, 14, 21, and 28 days following islet infusion.

Unmethylated INS DNA levels were measured 18 times, at baseline before MMTT and IVGTT and before islet infusion and then at 15 min, 30 min, 1, 3, 6, 12, and 24 h and at days 3, 7, 14, 21 and 28 post TPIAT. Unmethylated insulin DNA levels were assessed after that at 3 months, 1 year and 2–3-year visit.

Serum samples were collected and stored in separate aliquots at −80°C until assayed.

### Mechanistic Assays

#### Cytokines

Cytokine samples were tested by the Cytokine Reference Laboratory (CRL, University of Minnesota). Samples were analyzed for human specific IL-6, IL-8, IL-1α, IL-1β, MCP-1, TNFα, IL-10, and IP10 using the Luminex platform and performed as a multi-plex. The magnetic bead set (cat. # FCSTM18-08; Lot # 1598610) was purchased from R&D Systems, Minneapolis MN.

#### Serum Alpha-1 Antitrypsin

The sA1AT levels were processed by the M Health Fairview laboratory and sent to the Mayo Diagnostic Laboratory for analysis using Siemens Nephelometer II.

#### Unmethylated INS DNA

The unmethylated INS DNA assay used droplet digital polymerase chain reaction (ddPCR). Quick-cfDNA Serum & Plasma kits and EZ DNA Methylation kits (Zymo Research, Irvine, CA) were used for DNA purification and bisulfite treatment of the DNA. We measured the levels of INS DNA by droplet digital PCR targeting two methylation-sensitive sites of the human INS gene in positions +396 and +399 from the transcription start site (hg19_knownGene_uc021qcd.1 range = chr11:2181009–2182439). Each 25-uL reaction volume consisted of Droplet Digital PCR supermix (Bio-Rad Laboratories, Hercules, CA), 900 nM of primer, 250 nM of probe and 10-uL of sample. The mixture and droplet generation oil were loaded onto a droplet generator (Bio-Rad Laboratories), and the generated droplets were transferred to a 96-well PCR plate. The PCR reaction was run on a thermal cycler with a 10-min activation at 95°C, 40-cycle two-step amplification protocol (30 s at 95°C denaturation and 60 s at 58°C), and 10-min inactivation step at 98°C. The DNA content of the droplets was analyzed with a QX200 droplet reader (Bio-Rad Laboratories) and QuantaSoft analysis software (Bio-Rad Laboratories). Discrimination between droplets that did and did not contain the target (positive and negative, respectively) was achieved by applying a fluorescence amplitude threshold based on the amplitude read from the negative template control. For each sample, the ratio was calculated as the unmethylated counts divided by the sum of the unmethylated and methylated counts (U/[M + U])

### Statistical Methods

Characteristics of the three treatment groups (Table 1) were compared using one-way ANOVA for characteristics on continuous scales (e.g., BMI) and Fisher’s exact test for characteristics on categorical scales (e.g., etiology).

**TABLE 1:** Characteristics of the treatment groups, displayed as mean (SD) or N (%).

Characteristic	All participants	Control	Etanercept	A1AT	*p*-value
*n*	43	16	14	13	
Age, years	38.5 (12.0)	37.4 (12.1)	40.8 (12.0)	37.3 (13.1)	0.70
Sex (male)	17 (40%)	3 (19%)	4 (29%)	9 (75%)	**0.009**
White	40 (93%)	16 (100%)	11 (79%)	12 (100%)	0.051
Hispanic	4 (9%)	1 (6%)	1 (7%)	2 (17%)	0.66
BMI Pre-TPIAT (kg/m^2^)	24.9 (4.2)	24.1 (4.5)	24.7 (3.7)	25.8 (4.7)	0.58
Etiology					0.20
Genetic	20 (47%)	7 (44%)	4 (29%)	8 (67%)	
Obstructive	9 (21%)	2 (13%)	6 (43%)	1 (8%)	
Idiopathic	10 (23%)	6 (38%)	2 (14%)	2 (17%)	
Other	4 (19%)	1 (6%)	2 (14%)	1 (8%)	
Total IEQ (x 10^5^)	2.4 (1.3)	2.2 (1.4)	2.7 (1.3)	2.4 (1.3)	0.58
IEQ/kg	3,313 (1987)	3,341 (2,396)	3,800 (1899)	2,864 (1,460)	0.50
Intraportal IEQ/kg	3,046 (1766)	3,096 (2,172)	3,260 (1,592)	2,752 (1,455)	0.78
Total islet number (x 10^5^)	2.7 (1.4)	2.8 (1.6)	3.2 (1.5)	2.3 (1.1)	0.28
Islet number per kg body weight	3,851 (2,239)	4,329 (2,758)	4,485 (1964)	2,687 (1,140)	0.075
Tissue volume (mL)	14.1 (9.8)	13.5 (10.3)	18.5 (10.3)	10.4 (7.2)	0.10
All islets intraportal	31 (72%)	12 (75%)	6 (43%)	12 (100%)	**0.004**
Pre-Op labs					
Hba1c	5.3 (0.4)	5.3 (0.4)	5.2 (0.4)	5.3 (0.4)	0.51
ACRglu (ng/mL*min)	30.8 (18.9)	25.9 (12.5)	39.6 (26.8)	27.0 (10.7)	0.097
ACRpot (ng/mL*min)	8.3 (4.5)	7.2 (4.1)	9.2 (4.4)	8.7 (5.3)	0.47
AIRglu (mU/L*min)	472 (398)	382 (270)	632 (579)	398 (179)	0.18
AIRpot (mU/L*min)	176 (115)	158 (107)	186 (133)	187 (109)	0.74
AUC_glucose (10^4^ mg/dL*min)	1.38 (0.23)	1.40 (0.26)	1.35 (0.19)	1.34 (0.20)	0.73
AUC_C-peptide (ng/mL*min)	600 (408)	545 (432)	730 (502)	525 (230)	0.37

*P*-values are in bold for statistically significant differences.

For group comparisons according to diabetes outcomes and islet cell function (Table 2), we present unadjusted analyses using Fisher’s exact test (on insulin, yes/no) or one-way ANOVA (all other outcomes). We performed two sets of adjusted analyses, adjusting for IEQ/kg and for IEQ/kg and sex; the results did not change notably (data not shown). The adjusted analyses used logistic regression (on insulin, yes/no) or multiple linear regression (all other outcomes).

**TABLE 2:** Two-year outcomes.

	N	Control	Etanercept	A1AT	*p*-value
2 Year outcomes, *N (%) or mean (SE)*					
On Insulin	42	14 (87.50%)	9 (64.29%)	10 (83.33%)	0.32
Insulin dose (u/day)	40	13.0 (3.5)	16.8 (3.9)	16.1 (4.3)	0.75
Hba1c (%)	37	6.3 (0.4)	7.1 (0.4)	7.0 (0.4)	0.45
FSIVGTT, ACRglu (ng/mL*min)	32	5.53 (2.10)	7.96 (2.39)	5.77 (2.52)	0.72
FSIVGTT, AIRglu (Mu/L*min)	32	112.0 (37.6)	149.2 (42.8)	105.7 (45.2)	0.74
GPAIS, ACRpot (ng/mL*min)	31	1.38 (0.45)	2.03 (0.51)	2.60 (0.57)	0.25
GPAIS, AIRpot (mU/L*min)	31	34.3 (11.8)	47.4 (13.5)	56.9 (15.1)	0.49
MMTT, AUC_glucose (10^4^ mg/dL*min)	32	21,493 (1921)	18,040 (2,190)	21,846 (2,309)	0.40
MMMT, AUC_C-peptide (ng/mL*min)	32	165 (39)	276 (44)	235 (47)	0.18
Mean glucose on CGM, mg/dL	27	146.6 (11.7)	136.6 (14.0)	129.0 (15.9)	0.66
% Time >180 mg/dL	27	25.6 (7.5)	19.2 (9.0)	12.2 (10.2)	0.56
% Time <70 mg/dL	27	2.0 (0.8)	1.3 (0.9)	2.7 (1.1)	0.66

Analyses of cytokine profiles, sA1AT, and unmethylated insulin DNA included multiple time points per participant and used mixed linear models with the restricted-likelihood method, with fixed effects group, time (treated as a categorical factor), and their interaction; adjusted analyses added adjusters as fixed effects. The random effect was participant. For analyses of cytokine profiles, the outcome (dependent variable) was the common logarithm of the measured cytokine level. For the analysis of unmethylated insulin DNA, the outcome was the common logarithm of U/(U + M) + 0.026; the latter is the 2.5th percentile of non-zero U/(U + M) values and was added to all values so the log transformation could be applied to all values including zeroes.

All analyses used JMP (v. 16.1.0 Pro, SAS Institute Inc., Cary NC USA).

## Results

### Participant Characteristics

Table 1 describes the study’s participants. The age, BMI, and initial metabolic test results were comparable across the groups. However, the A1AT group had a significantly greater proportion of male participants (*p* = 0.004). Although the groups had comparable IEQ per kg body weight, the A1AT group had lower islet number per kg (not adjusted for islet size) (*p* = 0.045). The etanercept group was more likely to receive islet transplantation outside the liver (*p* = 0.003), which might be due to a trend towards higher volumes of infused tissue in this group (*p* = 0.08). When comparing only the intraportal IEQ/kg infused, all groups received a similar intraportal islet mass.

### Diabetes Outcomes and Islet Cell Function 2 Years Post TPIAT

**FIGURE 1:**
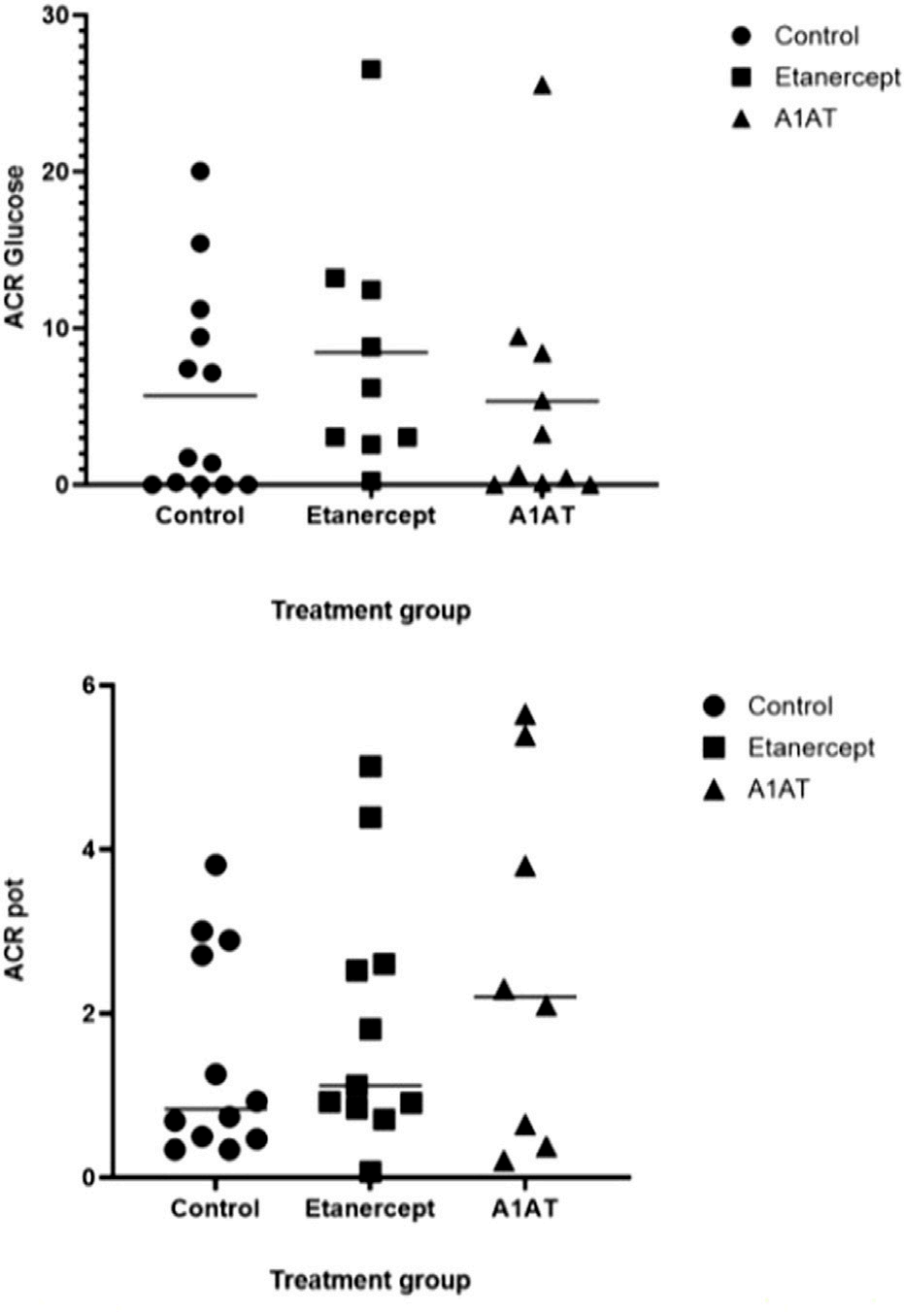
ACR glu and ACR pot at 2 years post TPIAT.

Insulin dose and glycemic control were similar in the three groups 2 years post-transplant (Table 2). Islet cell function assessed by MMTT, IVGTTT and GPAIS did not differ significantly between the three groups. The etanercept group had the best response to IVGTT (ACRglu and AIR glu) compared to control group and A1AT group (Figure 1) but this difference was not statistically significant (*p* = 0.72, *p* = 0.74 respectively).

### Cytokine Profiles

**FIGURE 2:**
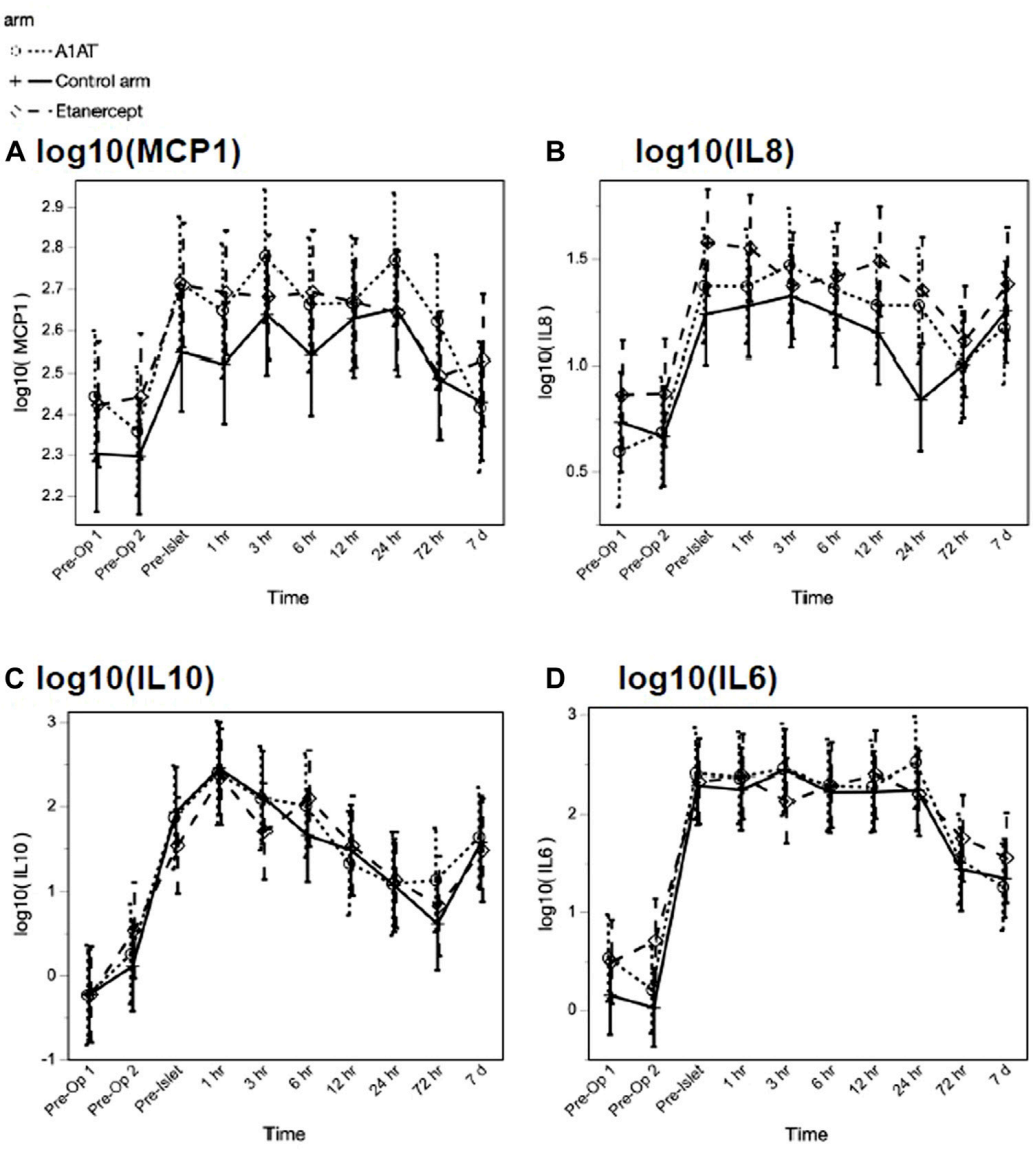
MCP1 (panel **A**), IL8 (panel **B**), IL10 (panel **C**) and IL6 (panel **D**) levels at different time points in the three groups.

Among the inflammatory cytokines assayed, only TNFα differed significantly by treatment, with highest levels in the etanercept group (*p* = 0.027). Other measured cytokines did not differ significantly by treatment group in their trajectories after islet infusion. As expected, we observed increases in inflammatory cytokines related to TPIAT, particularly for IL-6, IL-8, IL-10, and MCP-1, which all increased during TPIAT surgery, though notably these elevations occurred even before islet infusion (Figure 2).

**FIGURE 3:**
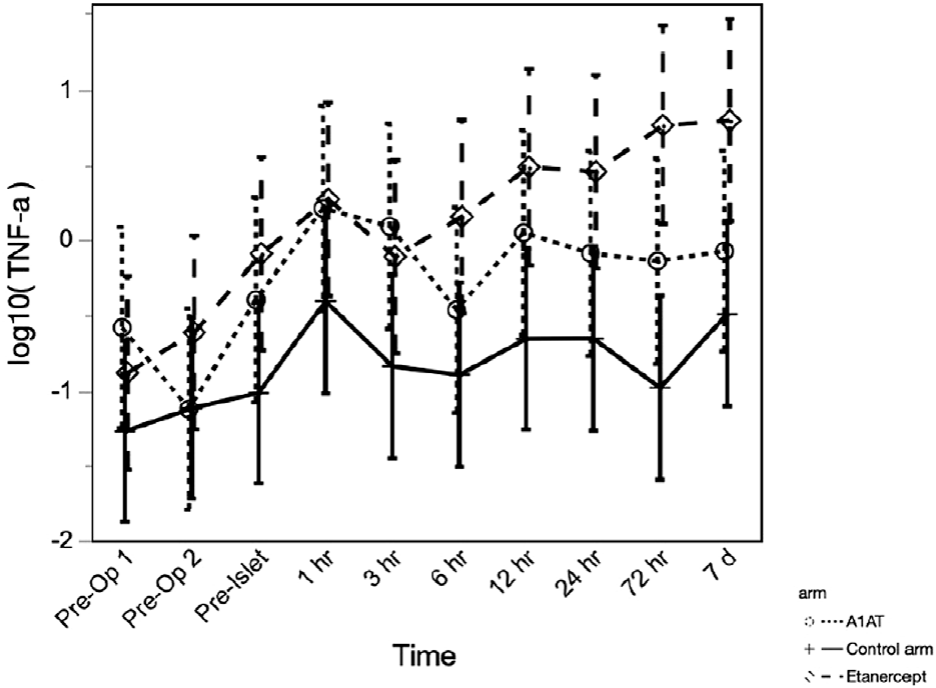
TNF-α level at different time points in the three groups.

The TNFα level was significantly higher in the etanercept group, compared to the A1AT group and the control group (*p* = 0.027) and continued to increase significantly with time (*p* < 0.0001) over the first 7 days post-TPIAT (Figure 3).

### Serum A1AT Level

**FIGURE 4:**
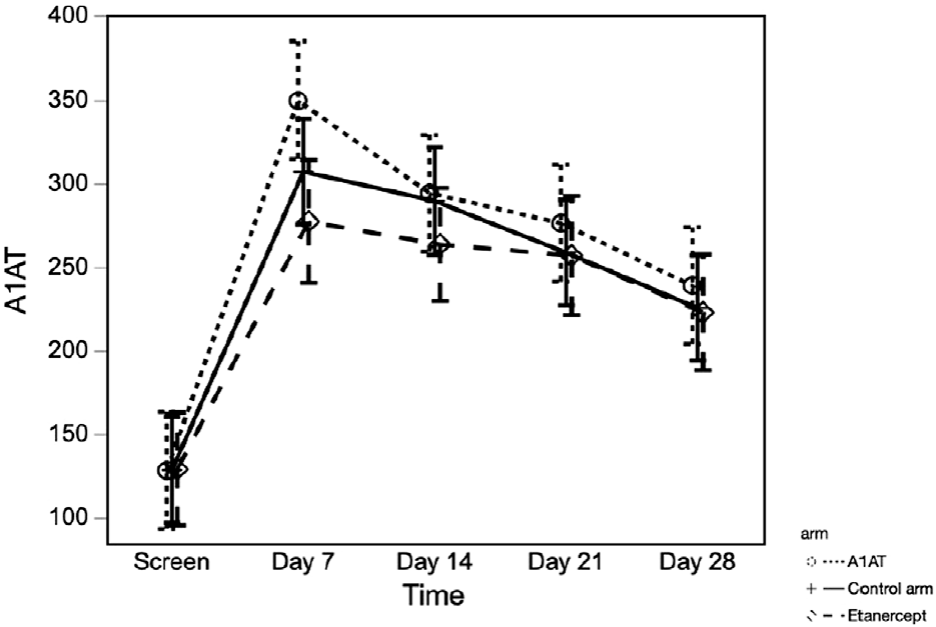
Serum A1AT level at different time points in the three groups.

Serum A1AT (sA1AT) levels increased significantly from pre-TPIAT baseline in all 3 groups (Figure 4)—including participants not treated pharmacologically with A1AT—with the peak level observed at 7 days post-TPIAT (*p* < 0.001). For all treatment groups, mean sA1AT was above the upper limit of normal, and remained above normal through 28 days post-TPIAT. Although the A1AT-treated group did not differ significantly from the other two groups when considering all times post-TPIAT (*p* = 0.32), the A1AT-treated group had a slightly higher peak at day 7 (*p* = 0.08 vs. control and *p* = 0.006 vs. etanercept).

### Unmethylated Insulin DNA

**FIGURE 5:**
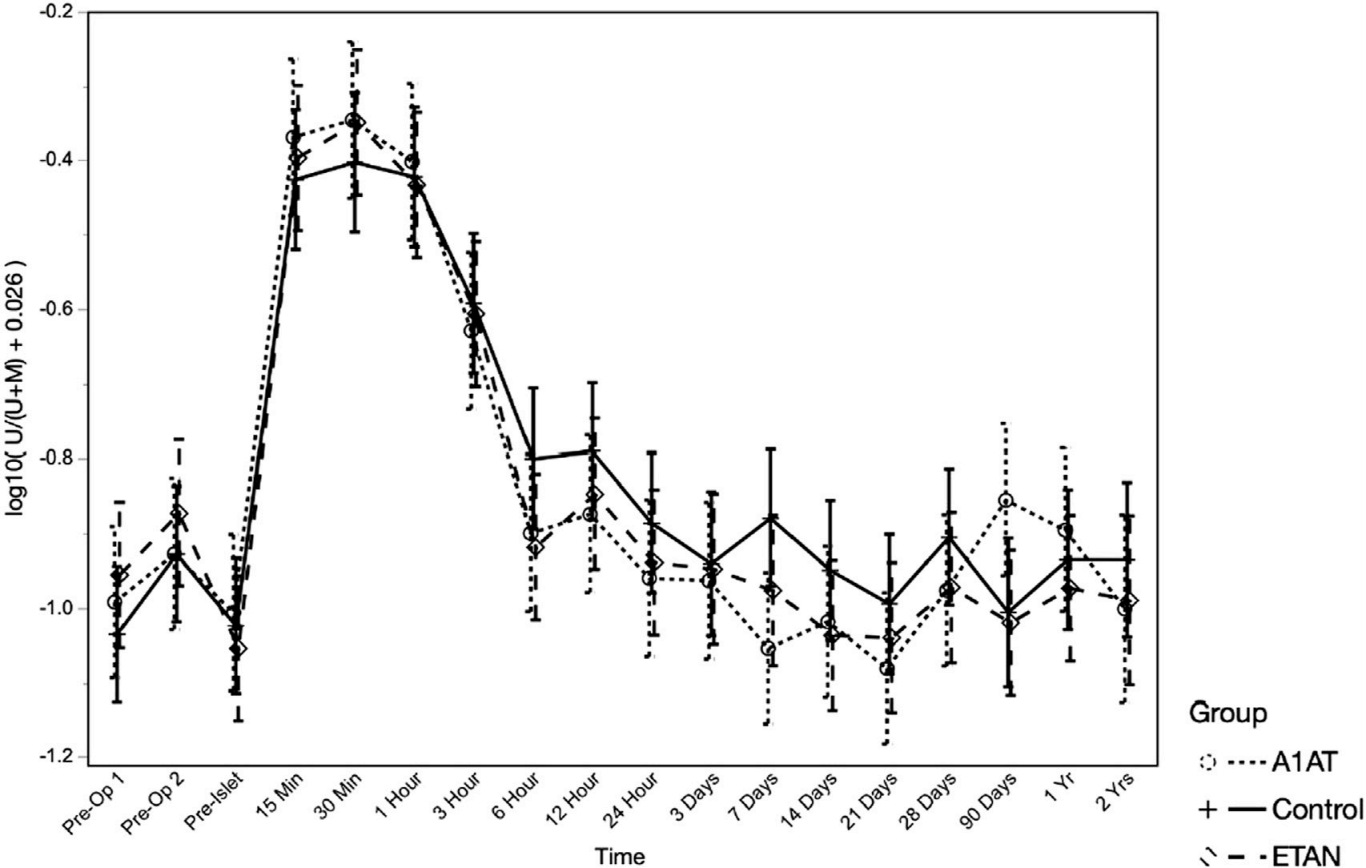
Unmethylated insulin DNA ratio at different time points in the three groups.

To assess whether islet loss differed between groups, we compared the log-transformed ratio of unmethylated insulin DNA to total insulin DNA (U/(U + M)) over time post-transplant, adjusted for islet mass (IEQ/kg) transplanted (Figure 5), adjusted for islet mass (IEQ/kg) transplanted. As expected, U/(U + M) increased significantly after TPIAT (*p* < 0.0001), with the highest values from 15 min to 1 h after islet infusion, consistent with known early islet loss. Higher IEQ/kg transplanted was associated with higher U/(U + M) (*p* = 0.013). When evaluating the trajectory of islet loss for the entire study period, from pre-TPIAT to 2 years post-TPIAT, the trajectories did not differ between groups (*p* = 0.66). However, a trend towards higher U/(U + M) was seen in the control group, vs. the etanercept and A1AT groups, from 6 h to day 28: in *post hoc* tests, controls were about 22% higher than participants treated with A1AT (*p* = 0.023) and 17% higher than those treated with etanercept (*p* = 0.067). This time frame is notable because it assesses islet loss during the drug treatment period, excluding the immediate islet loss that may be driven by islet damage during isolation.

### C-Peptide Level in the Peri-Operative Period

Peri-operative C -peptide level was highest at 1 h after islet infusion, reflecting early islet loss, and continued to decline until day 28. There was no significant difference between the three groups in C-peptide levels (*p* = 0.48).

## Discussion

This is the first randomized trial of either etanercept or A1AT treatment in patients undergoing TPIAT. We previously reported better insulin secretion at 3 months in the etanercept-treated group. In this second stage of our study, we evaluated long-term insulin secretion and glycemic outcomes, and early mechanistic markers of inflammation and islet loss. Overall, there was no evidence of clinically meaningful efficacy of short-term treatment with either etanercept or A1AT. Treated groups and untreated controls had similar inflammatory profiles and similar metabolic outcomes at 2 years.

We observed upregulation of endogenous circulating sA1AT in the control and etanercept groups, which has not previously been reported. Although the group receiving A1AT treatment showed slightly higher sA1AT soon after surgery, the difference in magnitude was relatively small. The finding of increased sA1AT in the non-treatment groups was unexpected—our intention was to measure the pharmacological effect of treatment with A1AT therapy. A1AT is produced in the liver as an acute-phase reactant in response to inflammation and its level can remain elevated for >1 week depending on the underlying trigger [19]. We suspect that ‘injury’ from the surgical trauma and islet infusion triggered secretion of higher levels of A1AT. Because we did not measure sA1AT level intraoperatively before islet infusion, we cannot determine whether elevation was precipitated by major surgery, rather than by islet infusion. We speculate that we did not see a benefit of pharmacologic treatment with A1AT because drug treatment only minimally enhanced already high endogenous levels.

At the 2-year follow-up, the three groups exhibited similar outcomes for insulin dependence, insulin dosage, HbA1c levels, and CGM data. Overall insulin independence rates were similar to historical outcomes. Also, the three groups did not differ in islet function as assessed using MMTT (mixed-meal tolerance test), IVGTT (intravenous glucose tolerance test), and GPAIS (glucose-potentiated arginine induced insulin secretion) tests. This is similar to what we reported previously at the 1 year follow up. Note that this study was designed as a pilot, and thus may be underpowered to observe small differences. However, the absence of a strong signal of robust benefit suggests a reduced likelihood of significant advantages associated with either A1AT or etanercept alone.

Consistent with our clinical outcomes, cytokine levels did not show any clear effect of treatment on the inflammatory cascade triggered by TPIAT. Cytokine levels (IP-10, IL-1α, IL-1β, IL-6, IL-8, IL-10, MCP-1, TNF-α) significantly increased from baseline after TPIAT. In our cohort this was present during surgery even *before* islet infusion, suggesting that at least some of this inflammatory cascade is triggered by the stress of the TPIAT surgery itself. This raises the possibility that improved outcomes could be achieved by better addressing this surgical inflammation before islet infusion. However, we did administer the first dose of etanercept or A1AT before the surgical procedure, and this single dose was not effective in mitigating the inflammatory response. Generally, inflammatory cytokines continued to be elevated 7 days post-TPIAT. Comparing the three groups’ cytokine profiles, the groups did not differ except in TNF-α, which was significantly higher in the etanercept group. This is likely a treatment effect of etanercept, which binds to TNF-α and prevents it from binding to its receptors. Elevated TNF-α levels have been reported in conditions treated with etanercept [20, 21].

These finding differ somewhat from prior work by Naziruddin et al, who reported that administering etanercept reduced IL-8, IL-6, and MCP-1 levels compared with controls [22]. However, that study was a retrospective, non-controlled study and in contrast, we have a randomized single-center study, which may avoid bias from time- or center-dependent effects. The same study found that combining IL-1 blockade with etanercept significantly suppressed elevation of IL-8, IL-6, and MCP-1 and led to better islet function as assessed by basal C-peptide, glucose and hemoglobin A1c. We did not attempt combination therapy in this pilot study, and multi-level blockade of inflammation might have better efficacy.

Our findings are also notably different from alloislet transplant studies in which *non-randomized* administration of etanercept is suggested to benefit long-term outcomes. There are, however, a few important distinctions between the allo- and autograft settings. First, although alloislet recipients do not undergo the major surgical procedure of pancreatectomy, in one small study directly comparing allo and autografts, the inflammatory response to alloislet infusion was much more pronounced, likely due to the immunologic contributions of HLA-mismatched tissue [23]. Second, alloislet recipients may have a pronounced TNF-alpha response to induction medications like anti-thymocyte globulin [24]. Lastly, the first dose of etancercept in the alloislet setting is administered intravenously. In our study we administered all doses, including the first dose, subcutaneously. This approach was chosen to mimic a protocol that could be used in the clinic, without need for an IND. Since all subsequent doses are administered subcutaneously in both our study and the alloislet setting, we would expect any impact from the different route of administering with the first dose to be limited to the first 3–7 days post-transplant. For these reasons, our results may not be directly extrapolated to the alloislet setting.

Our patients received etanercept intermittently between day 0 and day 21 or A1AT between day 0 and 28 after TPIAT. This selection of short-term treatments was based on the known limited duration of instant blood-mediated inflammatory reaction (IBMIR). The finding that etanercept resulted in better islet function 3 months post-TPIAT is promising and suggests that longer duration of treatment might have provided a more prolonged benefit. However, prolonging treatment duration could increase risks of side effects, as well as increasing financial costs. These considerations highlight the need to balance the potential benefits of longer treatment duration with the potential risks and resources required for such an approach.

Consistent with our prior reports, we did observe a robust rise in unmethylated INS DNA measures immediately after islet infusion, confirming that islet loss is universally occurring in these TPIAT recipients. Although the treatment groups did not differ overall, *post hoc* tests found an intriguing signal, from Hour 6 to Day 28, of lower levels of unmethylated INS DNA in the etanercept and A1AT groups combined, when adjusted for IEQ/kg. This analysis should be interpreted with caution, as it was undertaken *post hoc* based on observed patterns. In the context of a pilot study, however, it suggests a potential treatment effect, and may support the idea of treatment prolonged beyond Day 28.

One limitation of the current trial is that we cannot definitively identify a mechanism to explain the *lack* of therapeutic benefit of either etanercept or alpha-1 antitrypsin. There may be other mechanistic measures—such as local intrahepatic inflammatory and injury measures—that were not obtainable and which might have directly addressed reasons for failure of these investigational drug therapies. However, we hypothesize that the failure of these agents to favorably impact the post-TPIAT inflammatory response explains the lack of efficacy. It is important to note that this study was a pilot trial, so its statistical power may be limited in detecting small effects. Some data were missing due to missed visits or technical constraints, such as instances where intravenous access was lost. Some participants did not return for in-person testing at 2 years, which was partly compounded by interruptions in research visits necessitated by the COVID-19 pandemic, as well as hesitancy of some participants to travel by plane during the pandemic even after we were able to resume visits. In these cases, we collected data that could be gathered remotely, mainly insulin use and HbA1c levels.

In summary, this pilot study’s findings did not indicate a significant reduction in inflammation or improved islet engraftment with either etanercept or A1AT, as evidenced by similar cytokine profiles and markers of beta cell death among the three groups during the early post-transplant period. The etanercept group did, however, exhibit better islet function at 3 months post-TPIAT. Unfortunately, this improvement was not sustained at the 1-year and 2-year follow-ups. These results suggest that exploring different doses or extending the duration of etanercept treatment may lead to more prolonged effects that could potentially benefit patients undergoing TPIAT.

## Data Availability

The raw data supporting the conclusion of this article will be made available by the authors, without undue reservation.
